# Machine learning techniques for mortality prediction in critical traumatic patients: anatomic and physiologic variables from the RETRAUCI study

**DOI:** 10.1186/s12874-020-01151-3

**Published:** 2020-10-20

**Authors:** Luis Serviá, Neus Montserrat, Mariona Badia, Juan Antonio Llompart-Pou, Jesús Abelardo Barea-Mendoza, Mario Chico-Fernández, Marcelino Sánchez-Casado, José Manuel Jiménez, Dolores María Mayor, Javier Trujillano

**Affiliations:** 1 Servei de Medicina Intensiva, Hospital Universitari Arnau de Vilanova, Universitat de Lleida, IRBLleida, Avda Rovira Roure 80, 25198 Lleida, Spain; 2 Servei de Medicina Intensiva, Hospital Universitari Son Espases, Institut de Investigació Sanitària Illes Balears, Palma de Mallorca, Spain; 3 UCI de Trauma y Emergencias, Servicio de Medicina Intensiva, Hospital Universitario 12 de Octubre, Madrid, Spain; 4 Servicio de Medicina Intensiva, Hospital Virgen de la Salud, Toledo, Spain; 5 Servicio de Medicina Intensiva, Hospital Universitario Puerta del Mar, Cádiz, Spain; 6 Servicio de Medicina Intensiva, Complejo hospitalario de Torrecárdenas, Almería, Spain

**Keywords:** Intensive care unit, Machine learning techniques, Supervised algorithms, Traumatic patient, Mortality

## Abstract

**Background:**

Interest in models for calculating the risk of death in traumatic patients admitted to ICUs remains high. These models use variables derived from the deviation of physiological parameters and/or the severity of anatomical lesions with respect to the affected body areas. Our objective is to create different predictive models of the mortality of critically traumatic patients using machine learning techniques.

**Methods:**

We used 9625 records from the RETRAUCI database (National Trauma Registry of 52 Spanish ICUs in the period of 2015–2019). Hospital mortality was 12.6%. Data on demographic variables, affected anatomical areas and physiological repercussions were used. The Weka Platform was used, along with a ten-fold cross-validation for the construction of nine supervised algorithms: logistic regression binary (LR), neural network (NN), sequential minimal optimization (SMO), classification rules (JRip), classification trees (CT), Bayesian networks (BN), adaptive boosting (ADABOOST), bootstrap aggregating (BAGGING) and random forest (RFOREST). The performance of the models was evaluated by accuracy, specificity, precision, recall, F-measure, and AUC.

**Results:**

In all algorithms, the most important factors are those associated with traumatic brain injury (TBI) and organic failures. The LR finds thorax and limb injuries as independent protective factors of mortality. The CT generates 24 decision rules and uses those related to TBI as the first variables (range 2.0–81.6%). The JRip detects the eight rules with the highest risk of mortality (65.0–94.1%). The NN model uses a hidden layer of ten nodes, which requires 200 weights for its interpretation. The BN find the relationships between the different factors that identify different patient profiles. Models with the ensemble methodology (ADABOOST, BAGGING and RandomForest) do not have greater performance. All models obtain high values ​​in accuracy, specificity, and AUC, but obtain lower values ​​in recall. The greatest precision is achieved by the SMO model, and the BN obtains the best recall, F-measure, and AUC.

**Conclusion:**

Machine learning techniques are useful for creating mortality classification models in critically traumatic patients. With clinical interpretation, the algorithms establish different patient profiles according to the relationship between the variables used, determine groups of patients with different evolutions, and alert clinicians to the presence of rules that indicate the greatest severity.

## Background

Models for calculating the risk of death are used to assess the severity of the condition of traumatic patients. Classically, models for calculating the risk of death in traumatic patients have used two types of approximations. One approach consists of using physiological variables, which can define organic failures, which indicate a greater risk if their values are far from the levels defined as normality; and another approximation determines the severity according to the graduation of the anatomical lesions produced in the different body areas [1].

Although various studies have tried to take advantage of these two approaches – anatomical and physiological – there is still a need to look for systems that achieve better results and to obtain tools that can be used in healthcare practice [2].

Lesions are divided into groups associated with different anatomical areas, and their intensity can be assessed according to the Abbreviated Injury Scale (AIS) [3]. The physiological impact is assessed at the neurological, hemodynamic, and respiratory levels according to the Triage-Revised Trauma Score (T-RTS) [4].

In order to analyse the relationship between mortality, anatomical extension of the injury and its physiological repercussion, it is necessary to have large databases that include records of critically traumatic patients. The RETRAUCI (National Trauma Registry in ICU) study includes the participation of 52 ICUs in Spain and almost 10,000 patients [5].

Classification systems that use machine learning techniques (MLT) provide a global methodological vision and allow us to create multiple algorithms to achieve a more accurate result [6]. The current interest in MLT methodology applied to biomedical research is especially keen, and the development of these techniques requires adequate standardization and evaluation guidelines [7].

There are platforms that make it possible to work with multiple algorithms and that make the work more user-friendly and accurate in the construction and evaluation of results [8]. Among others, the WEKA (Waikato Environment for Knowledge Analysis) platform, developed by the University of Waikato, offers the possibility of using various algorithms and evaluating them with a single tool [9].

From a theoretical point of view, in the ideal conditions of a refined database with sufficient records, the No Free Lunch Theorem establishes that all the algorithms will optimize their results. With real data, however, this same theorem forces us to use different algorithms that will obtain different degrees of precision [10].

Some algorithms produce models with clinical interpretation, such as those based on classification trees, decision rules or Bayesian networks [11, 12]. Understanding the relationships between the variables that influence the classification of patients according to their severity offers us the possibility of understanding the different profiles of traumatic patients admitted to the ICU.

Our objective is to create different predictive models of the mortality of critically traumatic patients using machine learning techniques, to evaluate their performance and, if they can be interpreted, to evaluate relationships between the different types of variables included.

## Methods

### RETRAUCI database

RETRAUCI is an observational, prospective, and multicentre nationwide registry that currently includes 52 ICUs in Spain. The RETRAUCI database only collects traumatic patients admitted to the ICU. It has the endorsement of the Neurointensive Care and Trauma Working Group of the Spanish Society of Intensive Care Medicine (SEMICYUC) and currently operates in a web-based electronic format [13]. We include a five-year study period (2015–2019). Ethics Committee approval for the registry was obtained (Hospital Universitario 12 de Octubre, Madrid: 12/209). Due to the retrospective analysis of de-identified collected data, informed consent was not obtained. Hospital mortality was used as the outcome variable.

The variables collected were classified into several groups (Table 1).

**Table 1:** Risk factors associated with mortality. Description and attribute evaluation

Variable abbreviation	Type	Group	Description	Attribute evaluation (weight)
Age	N	Patient	Age in years	0.04767
Sex	C	Patient	Male / Female	0.00168
AHEAD	S	AIS	AIS scale for Traumatic brain injury (0–6)	**0.08977**
ANECK	S	AIS	AIS scale for neck injury (0–5)	0
AFACE	S	AIS	AIS scale for face injury (0–4)	0.00120
ATHORAX	S	AIS	AIS scale for thorax injury (0–6)	0.00949
AABDOM	S	AIS	AIS scale for abdomen injury (0–6)	0.00373
ASPINE	S	AIS	AIS scale for spine injury (0–6)	0.00380
AUPPEREXT	S	AIS	AIS scale for upper extremity injury (0–4)	0.00507
ALOWEREXT	S	AIS	AIS scale for lower extremity injury (0–5)	0.01186
AEXTERNAL	S	AIS	AIS scale for external and thermal injury (0–6)	0.00245
PointRF	S	T-RTS	Points of Respiratory Frequency (4–0)	0.02438
PointSBP	S	T-RTS	Points of Systolic Blood Pressure (4–0)	0.02855
PointGCS	S	T-RTS	Points of Glasgow Coma Score (4–0)	**0.09633**
MV	C	Status	Mechanical Ventilation (Yes/No)	0.05836
MASSIVEHEM	C	Status	Massive Haemorrhage (Yes/No)	0.01503
HEMODINAM	C	Failure	Hemodynamic failure (Yes/No)	0.04002
RESPIRATORY	C	Failure	Respiratory failure (Yes/No)	0.02438
AKIDNEY	C	Failure	Kidney failure (Yes/No)	0.02234
COAGULOP	C	Failure	Coagulopathy (Yes/No)	0.02234

*N* Numerical, *C* Categorical, *S* Scale, *T-RTS* Triage-Revised Trauma Score, *AIS* Abbreviated Injury Scale. Attribute evaluation (weight): Ranking of attribute with respect to Information Gain Attribute Evaluation method (in bold the most important)

First, we considered patient variables, such as Age and Sex. Variables were used that describe the importance of injuries by anatomical area according to the AIS model (2005 version) - severity levels ranging from 1(least severe) to 4–6 (most severe) [3]. The anatomical areas were head (AHEAD), neck (ANECK), face (AFACE), thorax (ATHORAX), abdomen (AABDOM), spine (ASPINE), upper extremity (AUPPEREXT), lower extremity (LOWEREXT) and external and thermal injuries (AEXTERNAL).

Also, we considered variables derived from the T-RTS, obtained from first medical attention before initiating resuscitation and/or mechanical ventilation, such as the Respiratory Rate (PointRF), Systolic Blood Pressure (PointSBP) and the Glasgow Coma Score (PointGCS), which range between 0 points (greater severity) and 4 points (normality) [4].

Next, patient treatment variables, such as the presence at the ICU of mechanical ventilation (MV) or the occurrence of a Massive Haemorrhage (MASSIVEHEM) requiring activation of the massive transfusion protocol, were also included [14].

Finally, variables that defined organic failures during the ICU stay: hemodynamic failure (HEMODINAM) indicated by the presentation of an SBP lower than 90 mmHg requiring the administration of volume, blood products, and vasoconstrictor support; respiratory failure (RESPIRATORY), indicated by the presence of PO2/FiO2 below 300; renal failure (AKIDNEY), indicated by an increase in creatinine > 1.5 times the initial, or 25% reduction in urine flow to less than 0.5 ml/kg/h for at least 6 h; and the presence of coagulopathy (COAGULOP), indicated by the prolongation of prothrombin and activated partial thromboplastin times in > 1.5 times the control or by levels of fibrinogen < 150 mg / dl or thrombocytopenia < 100,000 [13, 15, 16].

### Conventional statistics

Variable distribution was tested with the Kolmogorov–Smirnov test. The variable AGE did not meet the criteria of normality (*p* > 0.05). Variables are described as median (interquartile range) or as a percentage. For the comparison of survivors (A-ALIVE) and non-survivors (D-DIED), the Mann-Whitney test was used for continuous variables, and the chi-square test or Fisher’s exact test for categorical variables. A *p*-value of < 0.05 was taken as significant.

### Machine learning techniques

We used the WEKA Platform (version 3.8). We first use attribute selection methodology. Attribute selection is a technique used to extract the ranking of attributes and can help us by reducing the work of processing algorithms by discarding irrelevant variables. WEKA incorporates various attribute selection techniques. We use the Information Gain Attribute evaluation method. This method measures the significance of attribute by measure of information gain calculated with respect to target class and orders the variables according to their importance [17].

Second, we use WEKA’s EXPLORER module to determine the optimal parameters for each algorithm used. The parameters chosen were those that achieved the best performance values (see Algorithm evaluation section). A ten-fold cross-validation process system was used in all algorithms [18].

And third, using WEKA’s EXPERIMENTER module, run all algorithms 10 times, using repeated ten-fold cross-validation, to facilitate comparison of the predictive performance based on the different evaluation criteria that are available in WEKA [18].

### Algorithm selection

Of the multiple algorithms included in WEKA, we selected nine supervised algorithms classified in traditional and ensemble methodology. The first six are traditional models based on logistic regression binary (LR) functions, a neural network according to multilayer perceptron (NN), sequential minimal optimization (SMO), classification rules (JRip), classification trees (CT) and Bayesian networks (BN), respectively. We also included three models that use ensemble classification algorithms: adaptive boosting (ADABOOST), bootstrap aggregating (BAGGING), and random forest (RFOREST) [18]. With the WEKA EXPLORER module we select the optimal parameters of the different algorithms used.

For the LR model, we used a backward stepwise regression system with variable input with *p* <  0.05 and removal with *p* <  0.10. Odds ratios (OR) with a 95% confidence interval were calculated.

In the CT model, we used the J48 algorithm based on C4.5, obtaining a pruned tree [19]. The JRip algorithm uses a rule learner: Repeated Incremental Pruning to Produce Error Reduction (RIPPER) [20]. We limited tree growth (CT) and the number of rules (JRip), with a minimum of 20 instances.

For the BN, we used the TAN (Tree Augmented Network) variable relation search algorithm, which generates a graph that can be interpreted. This method does not assume the independence of the variables [21, 22].

The SMO implements John Platt’s sequential minimal optimization algorithm for training a support vector classifier [23]. In NN, we used the automatic mode for selecting the number of nodes in the hidden layer, with a learning rate of 0.3 and a momentum of 0.2 [24]. In RFOREST, we selected ten trees with the C4.5 algorithm [25]. In the rest of the algorithms (ADABOOST and BAGGING), we used the parameters that WEKA incorporates by default [18, 26].

### Algorithm evaluation

To evaluate the performance of the algorithms, we used the calculation of accuracy, specificity, precision, recall, F-measure, and the area under curve ROC (AUC).

A patient who dies can be classified correctly (true positive-TP) or incorrectly (false negative-FN) and a patient who survives can be classified correctly (true negative-TN) or incorrectly (false positive-FP). We define the evaluation indices as:

- Accuracy. The proportion of patients that are correctly labelled among the total number of patients. Accuracy = (TP + TN)/(TP + TN + FP + FN).
- Specificity. The proportion of patients predicted as survivors and are correctly identified. Specificity = TN/(TN + FP).
- Precision. The proportion of patients that are correctly predicted as dead among those labelled as dead. Precision = TP/(TP + FP)
- Recall (Sensitivity). The proportion of dead patients that are correctly labelled. Recall = TP/(TP + FN)
- F-measure. A measure that combine both Precision and Recall. F-measure = (2 x Precision x Recall)/ (Precision + Recall).”

WEKA’s Experimenter module, with ten repetitions, allows one to establish whether there are statistical differences between the evaluated properties of the algorithms using the paired T-Test (corrected) [18].

## Results

The RETRAUCI database enrolled 9790 patients in the 2015–2019 period. With 165 records, the data was not complete. The study group includes 9625 patients with a median age of 48 (33–64) years, 77.8% men and a hospital mortality of 12.6% (1212 patients).

Table 2 show demographic and clinical characteristics of patients according to mortality. Table 3 show values in the AIS model scale according to anatomical zone and mortality. It is observed that the factors without significant differences between survivors and non-survivors also have less weight (see last column of Table 1) according to WEKA’s attribute selection criteria. The ANECK and AFACE variables, with the lowest values, were not used in the construction of the models. The most important factors (PointGCS and AHEAD) are those associated with traumatic brain injury (TBI).

**Table 2:** Demographic and clinical characteristics of patients according to mortality

Variable	ALL ***N*** = 9625	SURVIVORS ***N*** = 8413	NON-SURVIVORS ***N*** = 1212	***p***-value
**Age (years)**	48 (33–64)	46 (32–61)	66 (47–78)	< 0.001
**Sex (% male)**	77.8	78.5	72.4	< 0.001
**PointRF**				< 0.001
**4**	84.1	86.4	68.2	
**3**	6.0	6.0	6.5	
**2**	1.9	1.2	6.9	
**1**	0.7	0.4	2.9	
**0**	7.3	6.1	15.5	
**PointSBP**				< 0.001
**4**	84.5	87.2	66.1	
**3**	6.3	6.0	8.6	
**2**	5.5	4.5	12.4	
**1**	0.5	0.3	1.8	
**0**	3.1	2.0	11.1	
**PointGCS**				< 0.001
**4**	66.5	72.1	27.8	
**3**	10.1	10.2	9.2	
**2**	9.2	8.6	13.2	
**1**	4.5	3.6	11.1	
**0**	9.7	5.6	38.8	
**MV**	48.0	42.8	84.1	< 0.001
**MASSIVEHEM**	6.0	4.5	16.5	< 0.001
**HEMODINAM**	34.5	30.1	64.9	< 0.001
**RESPIRATORY**	11.8	9.5	27.2	< 0.001
**AKIDNEY**	16.9	14.1	35.8	< 0.001
**COAGULOP**	15.9	12.8	37.4	< 0.001

Values expressed as percentages or median (Interquartile range), *RF* Respiratory frequency, *SBP* Systolic blood pressure, *GCS* Glasgow coma score. *MV* Mechanical ventilation. *p*-value: calculated using chi-square test or Mann-Whitney test

**Table 3:** Values in the AIS model scale according to anatomical zone and mortality

Variable	ALL N = 9625	SURVIVORS N = 8413	NON-SURVIVORS N = 1212	***p***-value
**AHEAD**				< 0.001
**0**	45.9	49.6	20.7	
**1**	3.8	4.1	1.7	
**2**	8.6	9.3	3.4	
**3**	16.0	17.0	9.4	
**4**	12.3	11.6	17.2	
**5**	13.2	8.4	47.1	
**6**	0.1	0.0	0.6	
**ANECK**				0.306
**0**	98.3	98.3	98.3	
**1**	0.4	0.5	0.4	
**2**	0.5	0.5	0.6	
**3**	0.5	0.5	0.3	
**4**	0.2	0.2	0.2	
**5**	0.1	0.0	0.2	
**AFACE**				< 0.001
**0**	79.2	79.0	80.7	
**1**	7.7	7.9	5.9	
**2**	10.1	10.2	9.6	
**3**	2.6	2.6	2.6	
**4**	0.4	0.3	1.2	
**ATHORAX**				< 0.001
**0**	50.8	49.6	59.1	
**1**	2.2	2.2	1.7	
**2**	9.2	9.7	5.9	
**3**	23.3	24.6	13.8	
**4**	10.7	10.4	12.9	
**5**	3.8	3.4	6.6	
**6**	0.1	0.0	0.1	
**AABDOM**				< 0.001
**0**	79.0	78.3	83.5	
**1**	0.9	1.0	0.3	
**2**	7.9	8.3	5.0	
**3**	6.9	7.3	4.5	
**4**	4.0	4.0	4.1	
**5**	1.3	1.1	2.4	
**6**	0.1	0.0	0.1	
**ASPINE**				< 0.001
**0**	72.5	71.9	76.7	
**1**	0.0	0.0	0.0	
**2**	17.3	17.9	13.2	
**3**	6.0	6.1	5.4	
**4**	1.9	1.9	1.2	
**5**	2.2	2.2	2.5	
**6**	0.2	0.1	1.1	
**AUPPEREXT**				< 0.001
**0**	74.3	73.0	83.6	
**1**	2.4	2.5	1.7	
**2**	20.5	21.6	12.7	
**3**	2.6	2.7	1.9	
**4**	0.2	0.3	0.1	
**ALOWEREXT**				< 0.001
**0**	70.4	69.3	78.0	
**1**	2.1	2.2	1.2	
**2**	10.0	10.5	6.0	
**3**	11.1	11.8	6.6	
**4**	4.6	4.9	2.5	
**5**	1.9	1.4	5.7	
**AEXTERNAL**				< 0.001
**0**	96.1	96.0	96.9	
**1**	2.6	2.8	1.2	
**2**	0.3	0.3	0.2	
**3**	0.3	0.4	0.1	
**4**	0.2	0.2	0.2	
**5**	0.5	0.3	1.3	
**6**	0.1	0.0	0.1	

Values expressed as percentages. *p*-value: calculated using chi-square test or Fisher’s exact test

Table 4 shows the results of the LR model. 13 variables are included. Do not include SEX or PointFR variables, and that there are three anatomical areas with some OR with values less than 1 (THORAX, LOWEREXT and UPPEREXT).

**Table 4:** Logistic Regression Binary model for mortality prediction

Variable	B coefficient	Standard error	OR (95% CI)	***p***-value
Age	0.051	0.002	1.05 (1.04–1.06)	< 0.001
AHEAD				
0	Reference			
1	0.091	0.281	1.10 (0.63–1.90)	0.746
2	0.114	0.202	1.12 (0.75–1.66)	0.573
3	0.150	0.147	1.16 (0.87–1.55)	0.307
4	0.911	0.135	2.49 (1.91–3.24)	< 0.001
5	2.011	0.128	7.47 (5.82–9.60)	< 0.001
6	4.394	1.200	80.99 (7.72–849.01)	< 0.001
ATHORAX				
0	Reference			
1	−0.256	0.305	0.77 (0.43–1.41)	0.401
2	−0.048	0.169	0.95 (0.68–1.33)	0.777
3	−0.513	0.124	0.60 (0.47–0.76)	< 0.001
4	−0.122	0.140	0.88 (0.67–1.16)	0.381
5	−0.036	0.195	0.96 (0.66–1.41)	0.855
6	−1.263	1.199	0.28 (0.03–3.12)	0.303
AUPPEREXT				
0	Reference			
1	0.107	0.306	1.11 (0.61–2.02)	0.727
2	−0.315	0.121	0.73 (0.57–0.92)	0.009
3	−0.544	0.295	0.58 (0.33–1.03)	0.065
4	−1.17	1.082	0.31 (0.03–2.57)	0.278
ALOWEREXT				
0	Reference			
1	−0.783	0.380	0.46 (0.22–0.96)	0.039
2	−0.559	0.169	0.57 (0.41–0.79)	0.001
3	−0.509	0.158	0.60 (0.44–0.82)	0.001
4	−0.931	0.251	0.39 (0.24–0.64)	< 0.001
5	0.400	0.228	1.49 (0.95–2.33)	0.079
PointSBP				
4	Reference			
3	0.316	0.152	1.37 (1.01–1.85)	0.038
2	0.356	0.148	1.43 (1.07–1.91)	0.016
1	0.815	0.375	2.26 (1.08–4.71)	0.030
0	1.325	0.185	3.76 (2.62–5.40)	< 0.001
PointGCS				
4	Reference			
3	0.063	0.142	1.07 (0.81–1.41)	0.658
2	0.392	0.137	1.48 (1.13–1.94)	0.004
1	1.072	0.157	2.92 (2.15–3.97)	< 0.001
0	1.810	0.122	6.11 (4.81–7.76)	< 0.001
MV	0.858	0.142	2.36 (1.93–2.89)	< 0.001
MASSIVEHEM	0.554	0.153	1.74 (1.29–2.35)	< 0.001
HEMODINAM	0.818	0.099	2.27 (1.86–2.75)	< 0.001
RESPIRATORY	0.541	0.106	1.72 (1.39–2.11)	< 0.001
AKIDNEY	0.641	0.098	1.89 (1.57–2.30)	< 0.001
COAGULOP	0.610	0.109	1.84 (1.49–2.28)	< 0.001

*RF* Respiratory frequency, *SBP* Systolic blood pressure, *GCS* Glasgow coma score. *MV* Mechanical ventilation, *OR* Odds Ratio, *CI* Confidence interval

**Fig. 1:**
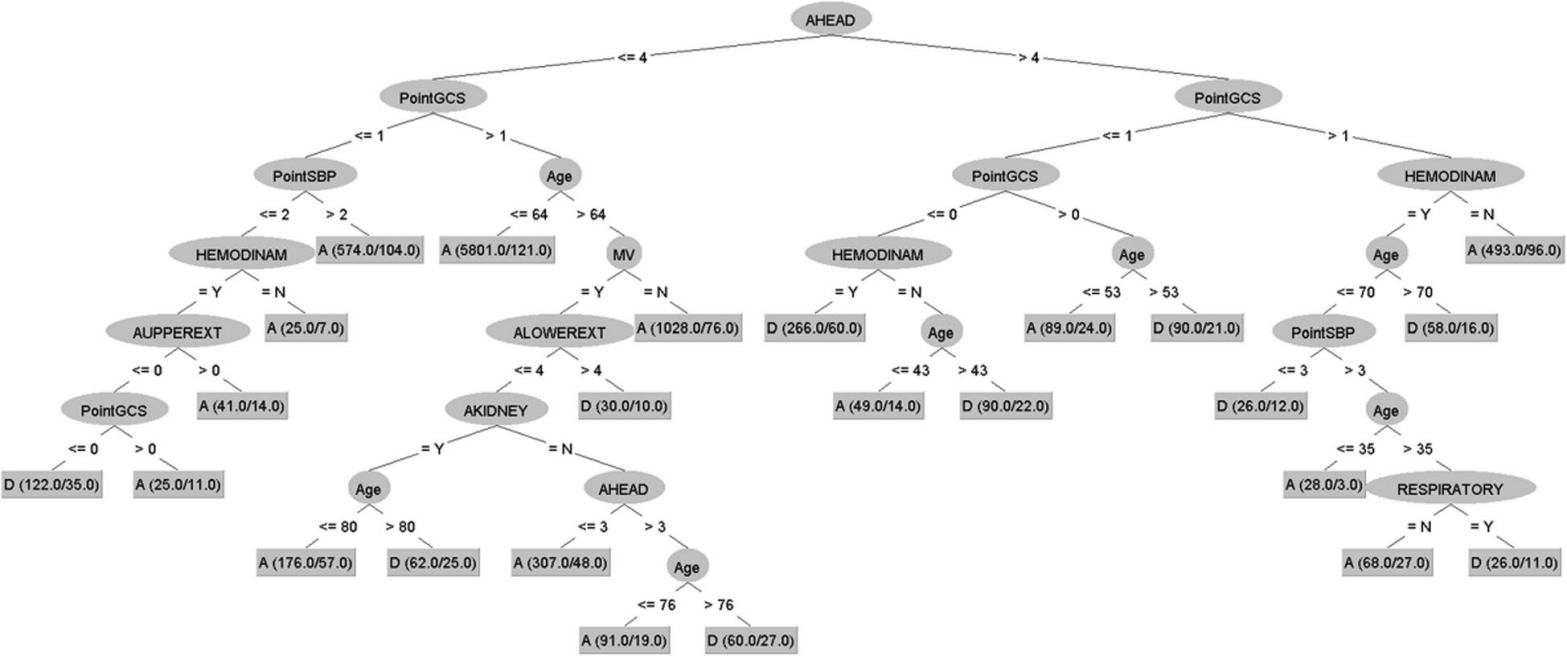
Mortality Classification Tree Model in Critically Traumatic Patients. A: Alive. D: Died

The CT algorithm (Fig. 1) generates 24 decision rules and uses those related to TBI as the first variables. A range of probability of death is obtained between 2.0 and 81.6%.

**Fig. 2:**
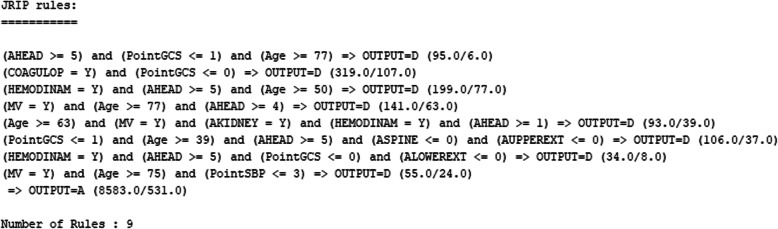
JRip-based classification rules. Output: Mortality. A: Alive. D: Died

The JRip algorithm detects eight classification rules (Fig. 2) that define the patients with the highest risk of mortality. The mortality rate ranges between 65.0 and 94.1%. Patients who do not comply with any of these rules have a lower mortality of 5.8%.

The NN model was established automatically with a hidden layer of ten nodes. The model is fully interconnected and requires 200 weights - 180 × 10 nodes in hidden layer and 10 × 2 in output layer - to be used for its interpretation.

**Fig. 3:**
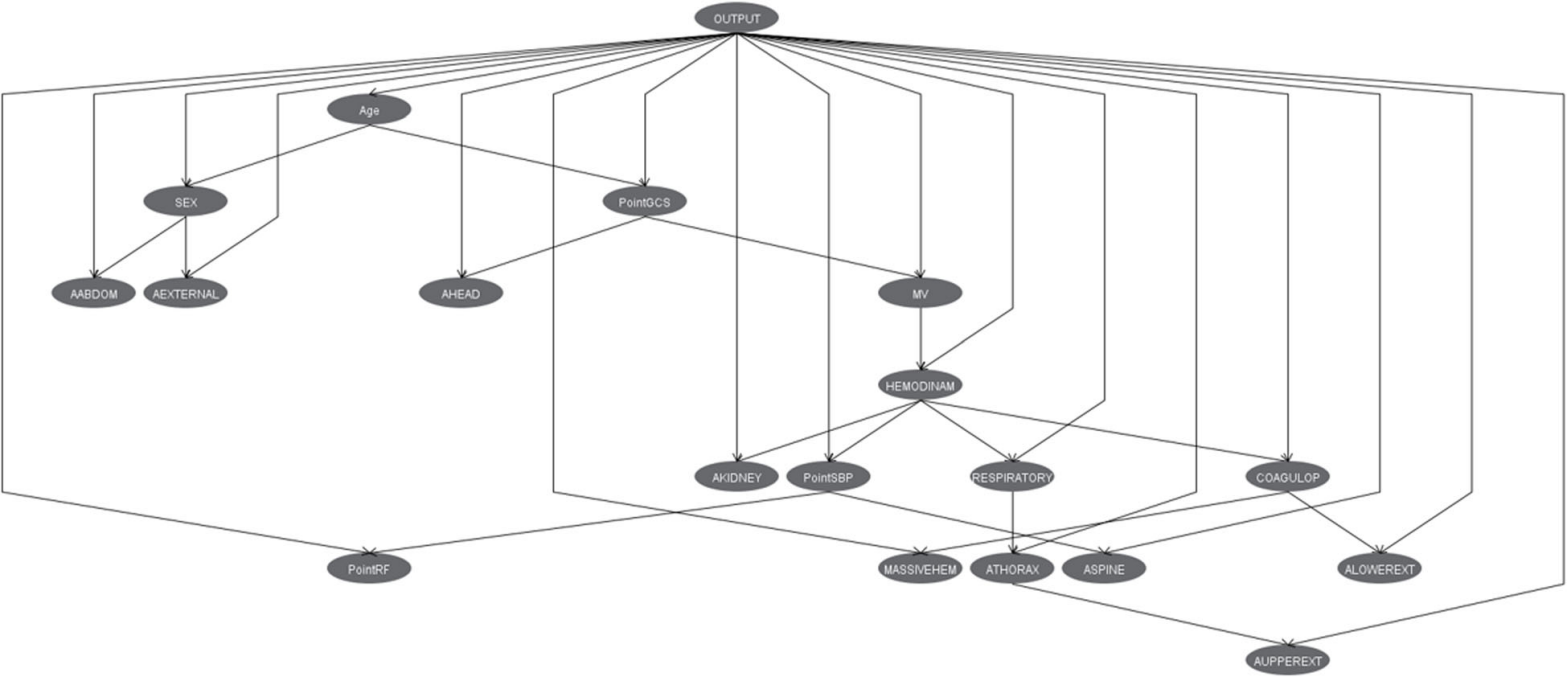
Bayesian network model (TAN) of mortality classification in critically traumatic patients. Output: Mortality

The BN model offers us a graph (Fig. 3) with which we can identify the relationships between the different factors, which, in turn, can help us identify different patient profiles. For example, the relationships are observed in the variables associated with TBI (AHEAD and PointGCS), hemodynamic failure with COAGULOP and AKIDNEY, respiratory failure with ATHORAX and AUPPEREXT and the relationship between ALOWEREXT injury with MASSIVEHEM and COAGULOP.

The performance data of the nine algorithms evaluated are shown in Table 5. No great differences were found in the precision measurements. All models obtained high values ​​in accuracy, specificity, and AUC, but obtained lower values ​​in recall. The highest precision was achieved by the SMO model, and the BN obtained the best recall, F-measure, and AUC.

**Table 5:** Performance properties of the 9 algorithms analysed

Algorithm	Accuracy	Specificity	Precision	Recall	F-measure	AUC
**LR**	0.901	0.970	0.685	0.459	0.623	0.912
**CT**	0.899	0.966	0.647	0.438	0.603	0.856
**JRip**	0.899	0.962	0.638	0.462	0.624	0.730
**BN**	0.894	0.955	0.603	**0.469**	**0.630**	**0.915**
**NN**	0.889	0.952	0.576	0.451	0.612	0.890
**SMO**	0.901	0.978	**0.710**	0.366	0.533	0.672
**ADABOOST**	0.892	0.971	0.630	0.337	0.500	0.891
**BAGGING**	0.902	0.968	0.668	0.444	0.609	0.910
**RFOREST**	0.901	0.964	0.650	0.460	0.623	0.905

*LR* Logistic regression model, *CT* Classification tree, *JRip* Repeated Incremental Pruning to Produce Error Reduction, *BN* Bayesian network, *NN* neural network, *SMO* Sequential Minimal Optimization, *ADABOOST* Adaptive boosting, *BAGGING* Bootstrap aggregating, *RFOREST* Random forest, *AUC* Area under ROC curve. In bold values with statistically significant differences

Despite using more complex algorithms, models with the ensemble methodology (ADABOOST, BAGGING and RandomForest) did not manage to increase the performance of the classification.

## Discussion

The availability of a database such as RETRAUCI gives us the opportunity to apply classification model methodology to stratify the risk of mortality and, therefore, establish the severity of traumatic patients admitted to the ICU [27]. Critical trauma patients have different characteristics from those who do not require admission to the ICU. Therefore, specific models for these patients should be developed using databases such as RETRAUCI [2, 28].

Classifying these patients in groups of different severity can help to prioritize the allocation of healthcare resources for the most seriously ill patients. There are several studies on the application of MLT in biomedical problems and in other aspects of critically ill patients [29, 30]. The WEKA platform enables us to carry out multiple classification models using a single tool [31].

In our results, the different algorithms have found certain common factors to be the most important in determining the risk of mortality. The most influential factors are those derived from TBI, both measured by anatomical involvement and by physiological repercussion. These results have already been studied in other studies on the severity of critical trauma patients [32]. The presence of an organic failure has also been shown to influence mortality [33]. Age is also a particularly important factor in the evolution of these patients [34].

In general, with some differences, the algorithms used have achieved similar levels of performance. The models have failed to classify the group of deceased patients with moderate recall values. Although this result coincides with other studies with different groups of patients, it requires us to continue searching for more precise algorithms [8].

The algorithms used have specific characteristics based on the clinical interpretation of the groups of patients with different severities and on the relationships of the different variables studied, which must be considered.

The LR results identify the variables that are independently associated with higher mortality. They also indicate that in critically ill patients, those with only more severe chest or limb injuries are a group which requires intensive surveillance, but which has a lower mortality rate among those admitted to the ICU [35].

The CT model serves to establish a hierarchy of variables and, through decision rules, establish different groups of patients according to their mortality rate. There are two large groups of patients: those with TBI and those without. It is also interesting to observe the different cut-off points for age according to each decision rule. Other models have been built into classification trees for both traumatic patients and other critical pathologies, and these have also found an increased risk of mortality associated with TBI [19, 36].

The JRip model shows easily interpretable classification rules [32]. In our work, it identifies the groups of patients with the highest mortality rate. This set of classification rules should become an alert system that identifies those patients with the highest risk of mortality early. In these groups of patients, the most important factors are advanced age, the presence of TBI and organic failure.

The BN-based model shows the relationship between the different factors studied. The relationship between factors dependent on head trauma can be appreciated. For example, the relationship between thoracic injury, respiratory involvement and upper extremity injuries is observed. On the other hand, the relationship between the presence of lower limb injuries (including the pelvis), coagulopathy, hemodynamic alteration and massive bleeding is observable. The study of these relationships is capable of differentiating groups of patients with different profiles of anatomical involvement and physiological repercussions. Traumatic patients admitted to the ICU share a critical process, but they express different forms of involvement that can be grouped into different profiles with specific characteristics in their severity and treatment. As in other works, the BN algorithm obtained better precision values [37].

The NN works with all possible relationships between the analysed factors. This characteristic has resulted in NN models obtaining the best classification results in other databases [24, 38]. In our case, it did not manage to improve the performance. In addition, the great complexity of its structure turned the model into a black box that It is difficult to interpret due to the large number of parameters to evaluate.

The ensemble algorithms, although more complex in their methodology, have been shown to obtain greater performance in other works. In our results, however, they also did not achieve greater performance values [39].

The ideal mortality risk calculation model must take two aspects into account. On the one hand, it must have the highest possible performance that can be achieved using more complex techniques in its calculation and/or include more predictor variables and their relationships. Complex models usually use specific programs for their use and are difficult to interpret due to the large number of parameters to be evaluated (for example NN). And, on the other hand, simpler models that have a great facility of clinical interpretation (R-TS and based on classification rules). We believe that they are not two divergent aspects, the creation of complex models can help to achieve that interpretable models improve their performance, for example, identifying variables and their relationships to incorporate them into models of clinical use.

## Limitations

Our work has several limitations. Other variables concerning the type or mechanism of the trauma, analytical or evolutionary, could have been included. Our objective required working with variables of anatomical involvement and physiological repercussion. More types of classification algorithms could also have been used [40, 41].

Also consider that an interpretable model may not learn ground truth relationships. For example, a causal interpretation of the Bayesian network in Fig. 3 would indicate that a person’s sex is caused by their age.

The incorporation of new records, which is carried out continuously in the RETRAUCI database, will allow the validation of the algorithms created and will incorporate more patients in the less numerous groups, such as in the neck lesions. The WEKA platform is a dynamic project that continuously improves the learning methodology by incorporating new algorithms and further automating the construction process [25].

## Conclusions

Machine learning techniques are useful for creating mortality classification models in critically traumatic patients. Even with some differences, the different algorithms achieved similar performance values. In addition, the algorithms that have a clinical interpretation help us to establish different patient profiles according to the relationship between the variables used and establish groups of patients with different evolutions, and some of the rules can even become alert systems to identify patients with the highest severity.

The models for classifying the severity of critically ill patients should have the common objective of determining the variables and their relationship in order to improve precision by establishing groups of patients with a greater probability of dying who could benefit from priority care that improves their survival.

## Data Availability

The datasets analysed during the current study are not publicly available due they are the property of the RETRAUCI project. Data are however available from the authors upon reasonable request and with permission of RETRAUCI project.

## Abbreviations

ADABOOST: adaptive boosting
AIS: Abbreviated Injury Scale
BAGGING: Bootstrap aggregating
BN: Bayesian networks
CT: Classification trees
FiO2: Fraction of Inspired Oxygen
GCS: Glasgow Coma Score
ICU: Intensive Care Unit
JRip: Classification rules
LR: Logistic Regression Binary
MLT: Machine Learning Techniques
MV: mechanical ventilation
NN: neural network
PO2: Partial pressure of oxygen
RETRAUCI: National Trauma Registry in ICU
RF: Respiratory rate
RFOREST: Random forest
SBP: Systolic blood pressure
SMO: Sequential minimal optimization
TBI: Traumatic brain injury
T-RTS: Triage-Revised Trauma Score
WEKA: Waikato Environment for Knowledge Analysis
